# A Recurrent Nonsense Mutation in NECTIN4 Underlying Ectodermal Dysplasia-Syndactyly Syndrome with a Novel Phenotype in a Consanguineous Kashmiri Family

**DOI:** 10.1155/2023/9999660

**Published:** 2023-10-04

**Authors:** Ghazanfar Ali, Sadia Sadia, Syeda Ain-ul- Batool, Zahid Azeem, Naheed Bashir Awan, Syed Akif Raza Kazmi, Zia- Ur- Rehman, Zeeshan Anjum, Fazal- Ur- Rehman, Abdul Wali, Kafaitullah Khan, Nasib Zaman, Muhammad Ayub, Muhammad Sajid, Noor Hassan

**Affiliations:** ^1^Department of Biotechnology, University of Azad Jammu and Kashmir, Muzaffarabad, Pakistan; ^2^Department of Biochemistry, Azad Jammu and Kashmir Medical College, Muzaffarabad, Pakistan; ^3^Department of Chemistry, Government College University, Lahore, Pakistan; ^4^Pir Mehr Ali Shah Arid Agriculture University, Rawalpindi, Pakistan; ^5^Department of Microbiology, Faculty of Life Sciences, University of Balochistan, Quetta, Pakistan; ^6^Department of Biotechnology, Faculty of Life Sciences and Informatics, BUITEMS, 87100, Quetta, Pakistan; ^7^Centre for Biotechnology and Microbiology University of Swat, Swat, Pakistan; ^8^Institute of Biochemistry, University of Balochistan, Quetta, Pakistan; ^9^Department of Pathology, College of Veterinary and Animal Sciences, Jhang Sub Campus of University of Veterinary and Animal Sciences Lahore, Lahore, Pakistan

## Abstract

EDSS1, a syndrome characterized by ectodermal dysplasia-syndactyly, is inherited in an autosomal recessive manner due to mutations in the NECTIN4/PVRL4 gene. Clinical manifestations of the syndrome include defective nail plate, sparse to absent scalp and body hair, spaced teeth with enamel hypoplasia, and bilateral cutaneous syndactyly in the fingers and toes. Here, we report a consanguineous family of Kashmiri origin presenting features of EDSS1. Using whole exome sequencing, we found a recurrent nonsense mutation (NM_030916: c.181C > T, p.(Gln61 *∗*)) in the NECTIN4 gene. The variant segregated perfectly with the disorder within the family. The candidate variant was absent in 50 in-house exomes pertaining to other disorders from the same population. In addition to the previously reported clinical phenotype, an upper lip cleft was found in one of the affected members as a novel phenotype that is not reported by previous studies in EDSS1 patients. Therefore, the study presented here, which was conducted on the Kashmiri population, is the first to document a NECTIN4 mutation associated with the upper lip cleft as a novel phenotype. This finding broadens the molecular and phenotypic spectrum of EDSS1.

## 1. Background

EDs are a genetically heterogeneous group of disorders characterized by developmental malformations of ectodermal structures, including nails, teeth, hair, or sweat glands. The prevalence of EDs varies depending on the subtype, with an approximate incidence of 7/10,000 cases live births [1]. To date, nearly, more than 200 forms have been described under the term ED [2]. EDs are further divided into two forms syndromic and nonsyndromic: nonsyndromic is pure; however, syndromic is associated with other defects including intellectual disability, eye diseases, skeletal defects, facial dysmorphism, and other systemic phenotypes [3, 4]. The identification of the molecular basis and pathogenesis of the increasing number of EDs has eased the development of a new classification system which combines both clinical and molecular data [2]. ED-syndactyly syndrome 1 (EDSS1; OMIM#613573) is a sporadic form of ED characterized by extremely thin or absent scalp hair, thin eyebrows, and eyelashes, palmoplantar hyperkeratosis, distant and conically shaped teeth, and enamel hypoplasia, along with partial cutaneous syndactyly in both fingers and toes. The etiology of ED-syndactyly syndrome 1 (EDSS1; OMIM#613573) has been attributed to genetic variations within the poliovirus receptor related 4 (PVRL4) gene, which has been more recently designated as NECTIN4 located on chromosome 1q23.1. NECTIN4 encodes a member of the nectin family, and its enhanced expression has been seen in adhering junctions of the suprabasal epidermis and the hair follicle, cultured keratinocytes, and in separating digits of the murine embryo [5]. The encoded protein contains one Ig-like V-type domain and two immunoglobulin-like (Ig-like) C2-type domains. The protein is involved in cell adhesion through trans-homophilic and heterophilic interactions. In multicellular organisms, cell-cell adhesion is crucial for the regeneration, ontogenesis, and maintenance of tissues and organs. All these functions are performed by different proteins. Dysregulation of genes associated with cell-cell adhesion proteins contribute significantly to the pathogenesis of many disorders such as, cancers, neuropsychiatric illnesses, and reproductive and sensory organs disorders along with other syndromic entities [6].

These cell-cell adhesion gene mutations have also been notably associated with a specific form of syndromic ectodermal dysplasias. For instance, the CDH3 gene mutation underlies the emergence of human juvenile macular dystrophy (HJMD; OMIM#601553), characterized by progressive central retinal degeneration and genetic hair loss [7, 8]. Similarly, mutations in the NECTIN1/PVRL1 gene located on 11q23.3 are linked with cleft lip/palate ectodermal dysplasia (CLPED1; OMIM#225060) [9]. Affected individuals from both EDSS1 and CLPED1 showed overlapping clinical features including thin scalp hair, eyebrows and eyelashes, tooth enamel hypoplasia, conical-shaped teeth, hypoplastic nails, palmoplantar keratoderma, and partial cutaneous syndactyly, whereas facial anomalies and cleft lip/palate are only seen in CLPED1 patients [10–12]. Till now, a limited number of mutations in the NECTIN4 gene have been reported in patients with EDSS1, and there is a lack of comprehensive genotype-phenotype associations. However, despite the diverse phenotypes of NECTIN4 mutations in EDSS1 patients and the lack of comprehensive genotype-phenotype associations, three clinical features, including cutaneous syndactyly, hair abnormalities, and dental anomalies, have been consistently observed in individuals with EDSS1, regardless of the ethnic background of patients [5, 11–15].

## 2. Methodology

### 2.1. Human Subject and Ethics Statement

The current study describes three generations of a consanguineous family from Neelum, Azad Jammu and Kashmir, Pakistan, who have clinical symptoms of EDSS1. Ethical approval to conduct this research was obtained from the Director of Advanced Study and Research (DSAR) Board of the University of Azad Jammu and Kashmir, Muzaffarabad, Pakistan. All ethical principles of the Declaration of Helsinki (October 2013) were followed in the handling of human subjects. Family elders were informed in detail about the purpose of the study in their local language, and all individuals taking part in this research gave informed written consent. A comprehensive medical report was obtained from the Department of Dermatology, Combined Military Hospital (CMH), Azad Jammu and Kashmir, Muzaffarabad, Pakistan.

### 2.2. DNA Extraction

Venous blood stored in EDTA-containing tubes was used to isolate genomic DNA using the phenol-chloroform method [16]. DNA quality was verified by using a photo spectrometer at 260 nm (UV-VIS SPECTROMETER/T60UV) and gel electrophoresis, respectively.

### 2.3. Whole Exome Sequencing and Variant Annotation

**Figure 1:**
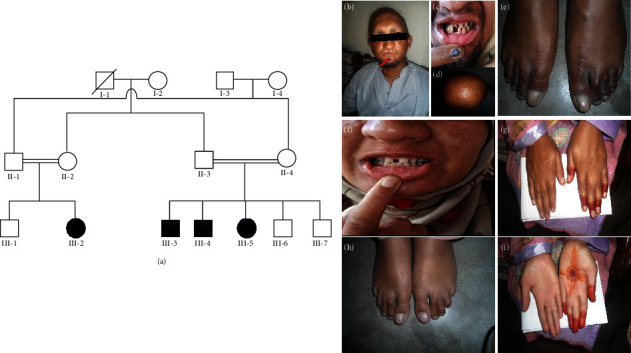
Pedigree and clinical manifestations of individuals affected with EDSS. (a) The pedigree displays the index patient used for whole exome sequencing, marked with (^*∗*^). All parents of the affected individuals have consanguineous relationships. (b–e) The clinical manifestations of individual (III-3) include hypotrichosis, with sparse or absent scalp hairs, eyebrows, and eyelashes, an upper lip cleft with spaced and pointed teeth, cutaneous syndactyly affecting fingers 3-4 on both hands and toes 2-3 on both feet, and a discolored nail palate. (f–i) The affected individual (III-5) displays clinical features consistent with EDSS.

Whole exome sequencing was performed on the DNA sample obtained from the affected proband (III-3) (Figure 1). The SeqCap EZ Exome v3 kit from Roche NimbleGen was used to enrich DNA libraries for whole exome sequencing, and the Illumina HiSeq 4000 (Illumina, San Diego, CA, USA) platform was used for whole exome sequencing. The mean depth of sequencing reads was maintained at 36x, ensuring adequate coverage of the target region, with each read covering approximately 94% of the region.

Low-quality reads were excluded by using Picard (https://broadinstitute.github.io/picard/), and then, the reads were mapped to the human reference genome (UCSC GRCh37/hg19) by using Burrows–Wheeler Aligner (http://biobwa.sourceforge.net/). Variants were called using Genome Analysis Tool Kit (https://software.broadinstitute.org/gatk/). Subsequently, the KGGSeq tool was utilized to perform a comprehensive set of annotations against reference sequences (Hg19). This included assessing frequency in publicly available databases, making conservative predictions, and predicting pathogenicity using available website-based tools for the detected variants [17].

### 2.4. Filtering and Prioritizing

Following the exclusion of all noncoding and synonymous variants, we further filtered out variants with minor allele frequency (MAF) ≥to 0.001 in the Exome Aggregation Consortium (ExAC) database (http://exac.broadinstitute.org/), the 1000 Genomes Project (http://www.internationalgenome.org/), and the Single Nucleotide Polymorphism Database (dbSNP) (https://www.ncbi.nlm.nih.gov/snp/). The retained variants with MAF <0.001 were then assessed for compound heterozygosity and homozygosity, with a focus on those consistent with the autosomal recessive inheritance pattern supported by the pedigree. A set of online bioinformatics tools including Polyphen2 (https://genetics.bwh.harvard.edu/pph2/), Fathmm (https://fathmm.biocompute.org.uk/), Mutation Taster (https://www.mutationtaster.org/), SIFT (https://sift-dna.org/sift4g), PROVEAN, and CADD (https://cadd.gs.washington.edu/score) were used to predict whether an amino acid substitution has an important biological effect on the protein structure and its functions. Subsequently, variants were selected by integrating the clinical features with information from relevant previously published literature, in accordance with the guidelines proven by the American College of Medical Genetics and Genomics (ACMG) [18].

### 2.5. Verification of the Candidate Region by Sanger Sequencing

Sanger sequencing was performed to validate the segregation of the candidate variant screened by WES among all the family members. A set of primer (forward: 5′TAATGGTGGCTGTCCCTCTCT 3′; reverse 3′ CACTCGTACTCGCCCTCATC 5′) was used for the amplification of the target region.

### 2.6. Protein Modeling and Functional Interactions

**Figure 2:**
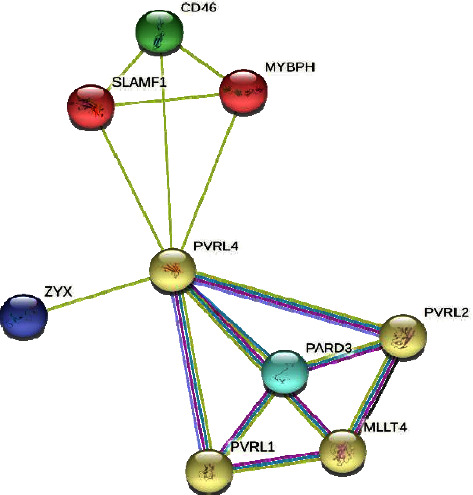
Interaction of NECTIN4/PVRL4 with other genes.

**Figure 3:**
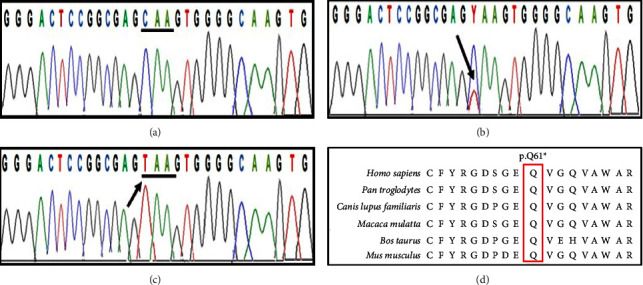
Electropherogram of the patient showing the site of nucleotide change. Black arrows indicate the position of nucleotide change (stop gain). (a) Wild type, (b) heterozygous carrier, (c) homozygous affected with a stop gain at position c.181C > T, and (d) comparison of the amino acid sequences of human NECTIN4 protein with orthologs from other species, indicating conservation of the p. Q61 residue across all species.

Utilizing the NCBI database (https://www.ncbi.nlm.nih.gov/), the amino acid sequence of NECTIN4 was obtained and used to create a 3D model of the protein through the application of I-Tasser software (Iterative Threading ASSEmbly Refinement) [19]. The produced structural models were subsequently visualized with PyMOL (https://www.pymol.org/), a renowned molecular graphics system. The functional interactions of NECTIN were evaluated using the string database (https://stringdb.org/) (Figure 2), while NCBI HomoloGene (https://www.ncbi.nlm.nih.gov/Homologene/) was utilized to investigate the conservation of amino acids across various orthologs (Figure 3(d)).

## 3. Results

### 3.1. Clinical Features

The family had four affected individuals including two males and two females. They had sparse and thin hair on the scalp and had sparse eyebrows and eyelashes. In addition, partial cutaneous syndactyly involving toes 2-3 and fingers 3-4, short fingers, and large palm size were also observed in the affected individuals. Conical teeth with pig-like shapes, enamel ridges, and pits characterized the dental features of all affected subjects, along with noticeable spacing. Aberrant sweating rates were evident across the hands, face, and scalp. A previously unreported clinical phenotype, an upper lip cleft, was observed in an affected individual (III-1) (Figure 1(a)). Detailed clinical features are given in Table 1.

### 3.2. Analysis of Exome Sequencing

An initial analysis of VCF of WES data revealed 71,630 variants. After filtering all synonymous and common variants with MAF threshold ≥0.001 in 1000 Genomes Project, ExAC, and dbSNP, 5,114 variants were selected for further analysis. Since the pedigree revealed an autosomal recessive inheritance pattern as shown in Figure 1(a), we searched the VCF file specifically for homozygous variants. The filtering process uncovered a previously reported homozygous nonsense variant (NM 030916: c.181C > T, p.(Gln61 *∗*)) in the second exon of the NECTIN4 gene. Details of the whole exome sequence coverage metric for the candidate subject (III-3) (Figure 1(a)) are presented in Table 2. Several pathogenicity prediction tools, including SIFT, Polyphen2, Mutation Taster, FATHMM, and CADD, were used to calculate the pathogenicity index of the reported variant. The results indicated that the variant was classified as deleterious with a CADD phred score of 35, indicating that it is among the top 0.1% of deleterious mutations in the human genome. To determine whether the variant was segregating with the disorder within the family, we conducted Sanger sequencing of the identified mutation.

The index variant (NM_030916: c.181C > T, p.(Gln61 *∗*)) segregated perfectly with the disease phenotype (Figure 3(c)). Amino acid residue p. Gln61 *∗* in the human NECTIN4 protein was found to be highly conserved among different species (Figure 3(d)).

## 4. Discussion

The nectin family is comprised of Ca2+-independent immunoglobulin-like cellular adhesion molecules, including nectins 1 and 4. These proteins play a crucial role in cell adhesion via homophilic and heterophilic interactions [23].

Any defects in cell-cell adhesion molecules may cause various types of ectodermal dysplasias [24, 25]. More specifically, mutations in NECTIN 1 and NECTIN4 cause cleft lip/palate ED (CLPED1; OMIM#225060) and EDSS1, respectively [5, 9, 24, 25]. The expression of NECTIN4 has been seen in the adherens junctions of keratinocytes of suprabasal epidermal layers in the interfollicular skin, the inner root sheath, and the shaft cortex of the hair follicle. In addition, the presence of NECTIN4 was also detected in the interdigital skin during embryogenesis [5]. In this study, we report a clinical and genetic investigation of a consanguineous family of Kashmiri origin, segregating EDSS1 in an autosomal recessive manner. Affected individuals displayed clinical features including sparse and very thin scalp hair, sparse mustache, very thin eyelashes and eyebrows, conical and widely spaced peg-shaped teeth, along with bilateral cutaneous syndactyly, palmoplantar keratoderma, flat discolored thickened hypoplastic finger and toe nails, and upper lip cleft Figure 1(b)–1(i).

Till now, total nine families with EDSS1 and only one family with EDSS2 have been reported around the world; these included families were from Pakistan [10–12, 15, 20], Algeria and Italy [5], Afghanistan [22], and Turkey [14, 21]. Using whole exome sequencing, we have identified a previously reported homozygous nonsense mutation p.(Gln61 *∗*) in the NECTIN4 gene. Earlier, this variant was reported by Raza et al. in [11] in another family of Kashmiri origin; however, upon investigation, it was confirmed that both families had no evidence of relationship. According to the human genome mutation database (HGMD Professional 2023.4) and literature, only eleven mutations have been reported in NECTIN4, which include three nonsense (p.Asp61 *∗*, p.Gln77 *∗*, and p.Arg55 *∗*), a frameshift (p.Pro304Hisfs *∗* 2), five missense (p.Pro212Arg, p.His83Tyr, p.Val242Met, p.Thr185Met, and p.Leu81Pro), an exon 2 deletion, and an apparent missense (p.Arg284Gln) inducing NECTIN4 splicing [5, 11, 14, 15, 22].

**Figure 4:**
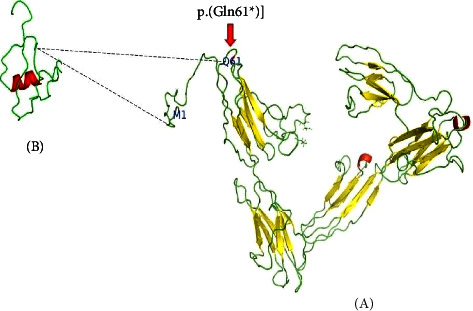
3D model of the NECTIN4 wild type and mutant protein. A: 3D model of NECTIN4 protein indicating the position of the start codon and a stop gain at position p.(Gln61 *∗*). B: mutant protein after stop gain.

The NECTIN4 gene encodes 510 aa nectin-4 protein. The protein has many domains counting a transmembrane domain, a cytoplasmic domain, three subdomains of immunoglobulin in the extracellular segment, and an N-terminal signal peptide. The candidate variant is situated in the V-type1 immunoglobulin-like segment of NECTIN4 (amino acids 32–144), as described earlier by Raza et al. [11], which results in the truncated protein [11] (Figure 4(a)).

Numerous tight junctional adhesion proteins complexes, desmosomes, and some other adhesive junctions play an important role in cell-cell adhesion, and the dynamic nature of this communication is vital for wound healing, tissue renewal, and establishing new cell contacts with other adjacent cells. Nectins (N-1 to N-4) are involved in cell-cell interaction through a calcium-independent adhesion mechanism and are considered as a supporting element for cell-to-cell adhesion by developing adhesive junctions (AJs). This phenomenon is based on heterophilic or homophilic interactions through Ig-like domains. Trans-heterophilic interactions are usually stronger than trans-homophilic interactions, and nectins engage in both homophilic and heterophilic interactions with other nectins or proteins on neighboring cells. These interactions trigger the creation of adherens junctions, which subsequently lead to the formation of tight junctions [26]. The regulation of the Rac1 gene activity is also linked to these nectins. In Rac1-deficient mice, interdigital webbing, defective skin, and severe hair loss have been observed, which are similar to the clinical manifestations seen in both EDSS1 and CLEPD1 patients. However, no disease-causing mutations in Rac1 have been identified in humans as of yet [22], and primary data for nectin in endothelium are scant.

In summary, the discovery of the “upper lip cleft,” a novel trait in EDSS1, is significant for future diagnosis and aids in developing a reference database specific to the Kashmiri population, as there is a lack of publicly available reference databases for identifying genetic mutations in this population. Moreover, findings could also lead to further clinical studies investigating the correlations between specific NECTIN4 mutations and clinical features, potentially improving the diagnosis and treatment of EDSS1 patients. Also, this study raises awareness of EDSS1 and related syndromes among researchers, healthcare providers, and the general public, facilitating prenatal diagnosis, genetic counseling, and timely interventions to improve the quality of life for affected individuals.

## Figures and Tables

**Table 1:** The clinical features observed in the current family and those reported in previous families.

Clinical features	Present study		[15]		Syed et al. 2015		[20]		[12]		[5]		[14]	[21]	[22]		Alshami 2015	
Age (years) at last examination	30–38		N/a		25–35		N/a		N/a		9–40		2	2.5	N/a		12–18	
Sex	2F	2M	9F	6M	2F	1M	3F	1M	7F	3M	4F	2M	1F	1F	2F	1M	1M	1F
Alopecia (P/C)	+(P)	+(P)	+(P)	+(P)	+(C)	+(C)	+(P)	+(P)	+(P)	+(P)	+(P)	+(P)	+(P)	+(P)	+(P)	+(P)	+	+
Hypodontia	—	—	—	—	+	+	—	—	—	—	—	—	—	—	—	—	—	—
Enamel hypoplasia	+	+	+	+	+	+	+	+	+	+	+	+	+	+	+	+	+	+
Spaced teeth	+	+	+	+	+	+	+	+	+	+	+	+	+	+	+	+	+	+
Discolored nail plate	+	+	+	+	+	+	+	+	+	+	—	—	—	—	—	—	+	+
Hyperkeratosis	+	+	+	+	+	+	+	+	+	+	—	—	—	+	—	—	+	+
Cutaneous syndactyly	+	+	+	+	+	+	+	+	+	+	+	+	+	+	+	+	+	+
Fingers	3-4	3-4	2-3-4	2-3-4	2-3-4	2-3-4	2-3-4	2-3-4	3-4	3-4	2-3, 3-4	2-3-4	—	2-3	2-3-4	2-3-4	3-4	3-4
Toes	2-3	2-3	2-5	2-3	2-3-4-5	2-3-4-5	2-3-4-5	2-3-4-5	2-3	2-3	2-3-4-5	2-3-4-5	2-3-4	3–5	2-3-4-5	2-3-4-5	2-3	2-3
Heat intolerance	+	+	+	+	+	+	+	+	N/a	N/a	—	—	+	+	+	+	+	+
Upper lip clift	—	+	—	—	—	—	—	—	—	—	—	—	—	—	—	—	—	—
Hearing disorder	—	—	—	—	—	—	—	—	—	—	—	—	—	_	—	—	+	+
Respiratory infection	—	—	—	—	—	—	—	—	—	—	—	—	—	—	—	—	—	—
Intellectual disability	—	—	—	—	—	—	—	—	—	—	—	—	—	—	—	—	—	—
Facial dysmorphism	—	—	—	—	—	—	—	—	—	—	—	—	—	—	—	—	—	—
Deformed pinnae	+	+	—	—	+	+	+	+	+	+	+	+	—	—	—	—	+	+
Limitation in joint movement	—	—	—	—	—	—	—	—	—	—	—	—	—	—	—	—	—	—
Learning difficulties	—	—	—	—	—	—	—	—	—	—	—	—	—	—	—	—	—	—
Sweating	Less	Less	Less	Less	Normal	Normal	Normal	Normal	Normal	Normal	Normal	Normal	Less	Less	Less	Less	Less	Less

**Table 2:** The process of identifying the disease-causing variant involved several filtering steps.

Filtration steps	Number of variants detected
Total variants detected in affected individuals (III-3)	71,630
The number of variants remaining after filtering out synonymous variants	35,466
The number of variants remaining after filtering out common variants with a minor allele frequency (MAF) of less than 0.01	5,127
Total homozygous variant (autosomal recessive)	20
Total homozygous missense variants detected	17
Total no. of frameshift variants	2
Total nonsense variant	1
Total variant segregated in all the affected individuals	1

## Data Availability

The data supporting the findings of this study will be available from the corresponding author upon request.
